# The third international stroke trial (IST-3) of thrombolysis for acute ischaemic stroke

**DOI:** 10.1186/1745-6215-9-37

**Published:** 2008-06-17

**Authors:** Peter Sandercock, Richard Lindley, Joanna Wardlaw, Martin Dennis, Steff Lewis, Graham Venables, Adam Kobayashi, Anna Czlonkowska, Eivind Berge, Karsten Bruins Slot, Veronica Murray, Andre Peeters, Graeme Hankey, Karl Matz, Michael Brainin, Stefano Ricci, Maria Grazia Celani, Enrico Righetti, Teresa Cantisani, Gord Gubitz, Steve Phillips, Antonio Arauz, Kameshwar Prasad, Manuel Correia, Phillippe Lyrer, the IST-3 collaborative group

**Affiliations:** 1 The IST-3 Co-ordinating Centre, Neurosciences Trials Unit, Bramwell Dott Building, Western General Hospital, Crewe Road, Edinburgh EH4 2XU, UK; 2 The University of Sydney, Discipline of Medicine, Westmead Hospital (C24), The University of Sydney NSW 2006, Australia; 3 Neurology Department, Sheffield Teaching Hospitals NHS Foundation Trust, Royal Hallamshire Hospital, Glossop Road, Sheffield S10 2JF, UK; 4 2nd Department of Neurology, Institute of Psychiatry and Neurology, Sobieskiego Str 9, 02-957 Warsaw, Poland; 5 Department of Experimental and Clinical Pharmacology, Medical University of Warsaw, ul.Krakowskie Przedmiescie 26/28, 00-927 Warsaw, Poland; 6 Department of Internal Medicine, Ullevaal University Hospital, NO-0407 Oslo, Norway; 7 Karolinska Institutet, Department of Clinical Sciences, Danderyd Hospital, SE-182 88 Stockholm, Sweden; 8 Service de neurologie, Cliniques universitaires Saint-Luc, Avenue Hippocrate 10, 1200 Bruxelles, Belgium; 9 Royal Perth Hospital, Wellington Street, GPO Box X2213, Perth, Western Australia, 6001, Australia; 10 Landesklinikum Donauregion Tulln, Neurologische Abteilung, Alter Ziegelweg 10, 3430 Tulln, Austria; 11 Ospedale Beato Giacoma Villa, Citta della Pieve, 06062-Perugia, Italy; 12 S C di Neurofisiopatologia, Azienda Ospedaliera di Perugia, Italy; 13 Division of Neurology, Dalhousie University and Queen Elizabeth II Health Sciences Centre, Halifax Infirmary, 1796 Summer Street, Halifax, Nova Scotia B3H 3A7, Canada; 14 Instituto Nacional de Neurologia, Insurgentes sur 3877, La Fama, 14269 Mexico DF, Mexico; 15 Department of Neurology, Neurosciences Centre, All India Institute of Medical Sciences, Ansari Nagar, New Delhi, 110029, India; 16 Neurology Department, Hospital Geral de Santo Antonio, Largo Prof Abel Salazar, 4050 Porto, Portugal; 17 Department of Neurology, University Hospital Basel, Petersgraben 4, CH-4031 Basel, Switzerland

## Abstract

**Background:**

Intravenous recombinant tissue plasminogen activator (rt-PA) is approved for use in selected patients with ischaemic stroke within 3 hours of symptom onset. IST-3 seeks to determine whether a wider range of patients may benefit.

**Design:**

International, multi-centre, prospective, randomized, open, blinded endpoint (PROBE) trial of intravenous rt-PA in acute ischaemic stroke. Suitable patients must be assessed and able to start treatment within 6 hours of developing symptoms, and brain imaging must have excluded intracerebral haemorrhage. With 1000 patients, the trial can detect a 7% absolute difference in the primary outcome. With3500 patients, it can detect a 4.0% absolute benefit & with 6000, (mostly treated between 3 & 6 hours), it can detect a 3% benefit.

**Trial procedures:**

Patients are entered into the trial by telephoning a fast, secure computerised central randomisation system or via a secure web interface. Repeat brain imaging must be performed at 24–48 hours. The scans are reviewed 'blind' by expert readers. The primary measure of outcome is the proportion of patients alive and independent (Modified Rankin 0–2) at six months (assessed via a postal questionnaire mailed directly to the patient). Secondary outcomes include: events within 7 days (death, recurrent stroke, symptomatic intracranial haemorrhage), outcome at six months (death, functional status, EuroQol).

**Trial registration:**

ISRCTN25765518

## Trial hypothesis

That intravenous recombinant tissue plasminogen activator (rt-PA) in a dose of 0.9 mg/kg (maximum 90 mg) administered to patients with acute ischaemic stroke, within six hours of symptom onset, increases the proportion of people alive and independent at six months.

## Background

### Acute ischaemic stroke is a major public health problem

Stroke is a common cause of death and serious disability. It has been estimated that stroke causes over four million deaths in the world each year, three million of these in developing countries, and thus is the second most common single cause of death (after ischaemic heart disease)[1]. In Europe alone, a quarter of a million people will become disabled after their first stroke each year. Although deaths from cerebrovascular diseases are declining in some parts of the world, rates are increasing in others (e.g. Eastern European countries)[2]. Even if age specific stroke incidence remains stable or falls slightly, as more people live into old age, the numbers of new cases of acute stroke per year may still rise.

### Reducing the burden of stroke: acute treatment of stroke is unsatisfactory

Despite better treatments to prevent stroke, stroke is likely to remain a common medical emergency for the next few decades. It has been estimated that in white populations about four fifths of all strokes are ischaemic and are usually due to sudden occlusion of extra or intracranial arteries by thrombus or embolic material. Once ischaemic stroke has occurred, treatment strategies aimed at restoring the normal arterial supply are likely to have the greatest impact on reducing the burden of stroke. Current treatment, however, remains unsatisfactory. Large randomised controlled trials have demonstrated that early (within 48 hours) treatment with aspirin for acute ischaemic stroke has only modest benefit (about 1% absolute reduction in death and recurrent ischaemic stroke[3]. This treatment effect is important as aspirin is an affordable and widely practicable treatment and is probably still underused. However, more effective treatments for acute stroke are needed.

### Thrombolysis for acute ischaemic stroke

Thrombolysis has been a standard treatment for acute myocardial infarction since the late 1980's. rt-PA was licensed for acute ischaemic stroke in the USA in 1996, but it was only granted a restricted licence for use in acute stroke by the European regulatory agency in 2003. Thrombolytic agents, by acting as plasminogen activators, break down the fibrin polymers of an acute thrombosis by converting plasminogen to plasmin, which in turn breaks down fibrin, releasing fibrin degradation products. The National Institute of Neurological Disorders and Stroke (NINDS) rt-PA Stroke Study group was the first trial to show evidence of benefit for thrombolysis in stroke patients[4]. Furthermore, the NINDS studies demonstrated that thrombolytic therapy for acute stroke was only feasible after major reorganisation of the assessment of patients with suspected stroke. Subsequent trials were less promising and the extra health service requirements of an effective stroke thrombolysis service resulted in very slow uptake of treatment [5-8]. The two main barriers to widespread use of thrombolysis were the remaining uncertainty over the effect of treatment in some categories of patient and the major investment in stroke service provision required for successful and safe implementation of treatment.

### Evidence on the effects of thrombolysis for patients with acute ischaemic stroke

The least biased and most precise assessment of the effects of a medical treatment is a systematic review of all the relevant randomised controlled trials. Two such reviews are available: the Cochrane systematic review[9] and the rt-PA Study Group pooled analysis [10]. The Cochrane systematic review included data from 8 randomised trials including 2955 patients. The rt-PA Study group pooled individual patient data on 2775 patients from 6 trials (NINDS part 1 and 2, ECASS-I; ECASS-II; and ATLANTIS Part A and B)[4-6,8,11]. The Cochrane review has the advantage that it includes all trials of rt-PA in acute stroke. The rt-PA Study group pooled analysis has the advantage that it enables the effect of treatment in specific subgroups to be explored, albeit in a smaller data set. Although these two independent reviews used different methods, they both came to broadly similar conclusions which strengthens their findings.

### Effect on deaths from all causes unclear

**Figure 1:**
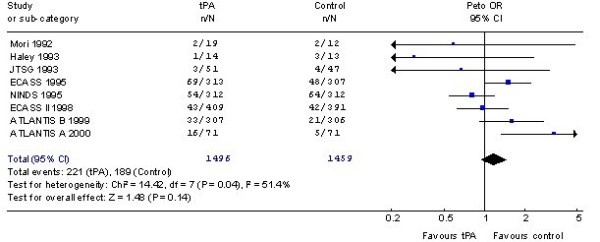
**Results of a systematic review of the randomised trials of thrombolysis with rt-PA in acute ischaemic stroke**. Effects on death at the end of follow-up. The estimate of treatment effect for each trial is expressed as an odds ratio (solid square) and its 95% confidence interval (horizontal line). The size of the black square is proportional to the amount of information available. An odds ratio of 1.0 corresponds to a treatment effect of zero, an odds ratio of less than 1.0 suggests treatment is better than control, and an odds ratio of greater than 1.0 suggests treatment is worse than control. The overall result and its 95% CI is represented by a diamond. (from Wardlaw *et al *[9]). Copyright Cochrane Collaboration, reproduced with permission.)

The Cochrane analyses show that rt-PA treatment was associated with a non-significant excess of deaths from all causes with rt-PA (OR 1.17, 95% CI 0.95 to 1.45 with a fixed-effect analysis) (see Figure 1). However, as there was significant heterogeneity, the overall estimate is difficult to interpret. Furthermore, the confidence intervals are wide and consistent both with a small reduction and a substantial excess of deaths. The heterogeneity may be due to many factors including time to treatment, dose of rt-PA, concomitant antithrombotic treatment and pre-treatment CT scan appearances.

### Effect of rt-PA on death or dependency

**Figure 2:**
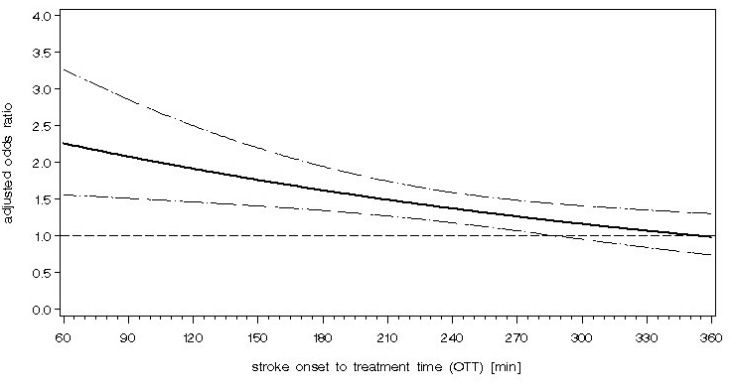
**The adjusted odds ratio of the chance of a favourable outcome** (modified Rankin score of 0–1, Barthel Index 95–100, NIHSS 0–1) at day 90 following thrombolysis with rt-PA by stroke onset to treatment time, derived from an analysis of individual patient data from the main randomised trials of rt-PA in acute ischaemic stroke. (from rt-PA Study group investigators [10], copyright Elsevier and the Lancet, reproduced with permission).

In the Cochrane review of all 8 rt-PA trials, treatment was associated with a significant reduction in the odds of being dead or dependent (OR 0.80; 95%CI 0.69 to 0.93). However, there was significant heterogeneity, and thus the estimate may not be reliable. The confidence interval was wide, and included the possibility that the benefit was very substantial or negligible. One factor that may explain some of the heterogeneity is the between-trial differences in stroke onset to treatment time. In the Cochrane review, this has been explored by analysing the results of rt-PA for early (<3 hours) and later (3–6 hours) treatment for trials that included patients in both time periods. These analyses did not demonstrate a statistically significant difference in treatment effect between the two time periods though there was a trend for earlier treatment to be associated with better outcome (< 3 hour treatment: OR 0.69; 95% CI 0.43 to 1.09, 3–6 hour treatment: OR 0.88; 95% CI 0.73 to 1.06). The results from the individual patient meta-analysis of the rt-PA Study Group provide an opportunity to explore this effect (and other factors) in much greater detail. The rt-PA Study Group analysis investigated the association between the odds of a good outcome (based on Rankin score, Barthel Index and NIH Stroke scores) and a series of potential clinical features including such factors as onset to treatment time, age, blood pressure, stroke severity and cerebrovascular risk factors. In a multi-variate analysis the main factor associated with a more favourable outcome was earlier treatment. The odds of a favourable outcome for those treated within 90 minutes was 2.81 (95% CI 1.75 to 4.50), declining to an odds ratio of 1.15 (95% CI 0.90 to 1.47) for those treated 271–360 minutes after stroke onset (see Figure 2).

The confidence intervals about the size of the early benefit within 3 hours are wide and there is certainly scope for substantial benefit from early treatment. Similarly, the width of the confidence intervals emphasises the lack of precision and the need for further data, even under 3 hours. On the other hand, the upper confidence interval suggests that worthwhile benefit from rt-PA may extend up to six hours (for those treated between 181 and 270 minutes from stroke onset, the odds of a favourable outcome was 1.40). The rt-PA study group commented that a large randomised controlled trial with over 5,000 patients (620 < 3 hrs and 4823 3–6 hrs) would be required to confirm or refute these findings. Nonetheless, whether given in routine practice, or as part of a trial, these data support the notion that 'time is brain' and every effort must be made to reduce time from onset to administration of thrombolytic treatment.

### Thrombolysis increases risk of symptomatic and fatal intracranial haemorrhage

In the Cochrane review, thrombolytic therapy with rt-PA was associated with a definite risk of fatal intracranial haemorrhage (OR 3.60, 95% CI 2.28 to 5.68, 2p < 0.00001) with no significant heterogeneity. The rt-PA Study Group investigators assessed the effect of several clinical factors: time to treatment, age, and stroke severity on the risk of intracranial bleeding. Treatment with rt-PA was the only independent predictor. Thus, at present, there are insufficient data available to guide clinicians on the factors that influence the occurrence of this most important side effect of treatment.

### Key unanswered questions about thrombolytic therapy for ischaemic stroke

#### What is the 'time window' for thrombolysis?

The current data suggest that the time window for treatment with thrombolysis may extend out to 6 hours from stroke onset. How long is the time window for effective treatment? Does the time window vary with patient factors? If treatment is effective up to six hours from stroke onset, a much larger number of patients would be eligible for treatment.

#### What is the effect of thrombolysis in older patients?

Only 42 patients aged over 80 years old have been included in the rt-PA trials to date (mainly as a consequence of the 80 years age limit in the ECASS studies). About a fifth of patients with stroke are aged 80 years or older, and this under-representation of older people represents a major gap in knowledge. Although the risk/benefit ratio of rt-PA might become less favourable with increasing age because of a higher risk of adverse events, the higher risk of a poor outcome without treatment could make treatment worthwhile for some older individuals. This can only be established by the inclusion of older people in further randomised trials. IST-3 will therefore have no upper age limit.

#### What is the effect of thrombolysis on overall survival?

The effect of rt-PA on deaths from all causes is unclear. There is a non-significant trend to an excess of deaths. Clinicians would be reassured if thrombolysis was shown to have no net detrimental effect on overall survival. If further trials confirmed that thrombolysis increased the risk of death, patients might still consider having the treatment, if those who survived the treatment had a much greater chance of being free of disability.

#### What predicts fatal intracranial haemorrhage?

Intracranial haemorrhage is the major risk of treatment and the current trial data cannot reliably identify independent risk factors, other than choice of agent, to predict bleeding. Even the more statistically powerful individual patient meta-analysis from the rt-PA Study Group Investigators was unable to identify any clinically relevant risk factors for cerebral bleeding (other than use of the thrombolytic agent itself). Yet there is a widespread belief that clinical factors do influence risk. Many factors could influence the risk of bleeding (and hence, the potential benefits of thrombolysis) and the most important factors to explore further include: age, prior antiplatelet therapy; stroke severity; stroke subtype, whether the infarct is 'visible on CT' or not and time to treatment. Reliable data on these factors will only emerge from further randomised controlled trials, as the current systematic reviews have been unable to clarify the role of these factors.

#### What pre-treatment CT scan appearances predict response to treatment?

Pre-treatment scans are obligatory to exclude intra-cranial haemorrhage. However, among patients with ischaemic stroke, certain features on the pre-treatment CT scan might predict the outcome of treatment. The extent and severity of any ischaemic changes on CT scanning might also provide additional prognostic information to time from stroke onset. Specific neuro-radiological features, such as the dense artery sign, might predict lack of response to treatment. Other features, such as extensive white matter change, may help identify patients at high risk of major intracranial haemorrhage with thrombolysis. Some previous analyses of pre-and post-treatment CT scans in the completed thrombolysis trials were not completely blind to treatment (and scan sequence) and the bias introduced may have over-emphasised the importance of some features.

### Current clinical practice

The lack of data and clinical uncertainty about the effects of thrombolysis for acute ischaemic stroke has led to divergent expert opinion [12-14], and as a result of this lack of consensus, the use of rt-PA is very variable [15,16]. Whilst there is strong support for the increased use of thrombolysis from many neurologists[17,18], other specialities and Emergency Medicine (EM) specialists have been more cautious[19,20]. The American Emergency Physicians statement stated: 'The challenge to those who are critical of this statement is to convince the EM community as was done for MI that this should be the standard of care. It may be difficult to do this without further research.' There is also debate about the criteria for selecting patients for thrombolysis[21]. Donnan stated: 'Clearly the view [on the indication for treatment] differs from physician to physician, country to country, and continent to continent[12]. The recent scientific statement from the American Heart Association emphasised that carefully selected patients who can be treated within 3 hours should be considered for treatment with rt-PA but 'caution should be exercised' for those with severe strokes (NIHSS > 22)[22]. However, the reanalysis of the NINDS trials from the rt-PA Acute Stroke Treatment Review Panel has demonstrated that patients with severe stroke (NIHSS > 20) in the NINDS trials had an absolute benefit of about 4–5% in independent survival, which is less than in stroke of moderate severity, but is still worthwhile. A Cochrane systematic review and an NHS Health Technology Assessment both concluded that rt-PA is promising, but further large-scale controlled trials were needed before the place of this treatment in routine clinical practice could be determined[9,23].

The philosophy of the IST-3 collaboration is therefore that only data from large-scale randomised trials comparing rt-PA with control can dispel these uncertainties. Such uncertainty might lead to many patients being denied an effective therapy and others being treated in error. A positive and ethical approach to take, in the current environment of uncertainty and differing expert opinion, is to enrol many thousands more patients in further randomised controlled trials. Furthermore, if IST-3 demonstrates that intravenous rt-PA can be given safely and effectively following an appropriate clinical assessment and urgent Computerised Tomographic (CT) scanning in a wide variety of emergency hospitals, treatment could be made more widely – and equitably – available to those that might benefit (and not, as at present, to the few who have access to the currently limited number of highly specialised stroke centres).

## Design

### Approval to start

Hospital centres in IST-3 must have the approval of the national co-ordinator before applying for ethics approval. Appropriate local Ethics Committee approval must be sought for each participating hospital. Proof of such approval must be sent to the trial office before recruitment can be started in each centre. The trial must be run according to local procedures and law.

### Trial centre requirements

A number of guidelines have stated thrombolysis should only be considered if the patient is admitted to a specialist centre with appropriate experience and expertise[22,24]. Hospitals participating in IST-3 should have an organised acute stroke service. The components of effective stroke unit care have been identified[25], so the service should be configured along those lines and also meet local standards and guidelines. In brief, the facilities (details of these requirements are specified in the separate operations manual) should include:

• Written protocol for the acute assessment of patients with suspected acute stroke to include interventions to reduce time from onset to treatment.

• Immediate access to CT or MR brain scanning (preferably 24 hours a day).

• A treatment area where thrombolysis may be administered and the patient monitored according to trial protocol, preferably an acute stroke unit.

### Eligibiity

Patients with mild, moderate or severe strokes are potentially eligible if the following criteria are met:

### Inclusion criteria

• Symptoms and signs of clinically definite acute stroke.

• Time of stroke onset is known and treatment can be started within six hours of this onset.

• CT or MRI brain scanning has reliably excluded both intracranial haemorrhage and structural brain lesions which can mimic stroke (e.g cerebral tumour)

### Exclusion criteria

• The patient has previously been randomised in IST-3

• Major surgery, trauma (e.g. major fall at time of stroke) or gastrointestinal or urinary tract haemorrhage within the previous 21 days. Arterial puncture at a non-compressible site within the previous 7 days.

• Any known defect in coagulation (e.g. currently on oral anticoagulants with an INR > 1.3 **OR **current treatment with heparin [unless APPT within normal laboratory limits] **OR **treatment with low molecular weight heparin or heparinoid **OR **treatment with ximelagatran).

• Known defect of clotting or platelet function (but patients on antiplatelet agents can be randomised).

• The patient is female and of childbearing potential (unless it is certain that pregnancy is not possible) or breast feeding.

• Hypo- or hyperglycaemia sufficient to account for the neurological symptoms; the patient should be excluded if their blood glucose is < 3.0 or > 20.0 mmol/L ('stick testing' is a sufficiently accurate test for this purpose).

• Symptoms considered likely to resolve completely within the next few hours (i.e. a TIA)

• Patient has had a stroke within the previous 14 days or has had treatment for acute ischaemic stroke with thrombolytic therapy within the past 14 days.

• Patient was already dependent in activities of daily living before the present acute stroke

• Patient has other life threatening illness (e.g. advanced cancer) likely to lead to death within a few months.

• Likely to be unavailable for follow-up e.g. no fixed home address.

• Patient has Systolic Blood Pressure < 90 mm Hg or > 220 mm Hg or Diastolic Blood Pressure < 40 mm Hg or > 130 mm Hg

### High blood pressure (BP) before randomisation

A persistently high blood pressure can be associated with a poor outcome after stroke[26], though high pre-treatment blood pressure was not an independent predictor of symptomatic intracranial haemorrhage with rt-PA[27]. Some patients with high blood pressure (i.e. systolic BP > 185 mm Hg and/or diastolic > 110 m Hg) can therefore be treated with rt-PA[22]. The randomisation system will only accept patients with systolic BP between 90–220 mm Hg and diastolic BP between 40–130 mm Hg. Although these data provide some guidance, the decision about whether or not to include a patient with persistently high levels of blood pressure in the trial must rest with the physicians' judgement.

### Uncertainty principle (absence of proof)

Further inclusion and exclusion criteria are not specified precisely but are guided by the uncertainty principle (or absence of proof for that particular patient). If, for whatever reason, the clinician is convinced that a patient fulfilling the above criteria should be treated, the patient should be treated with rt-PA and **NOT **randomised. If the clinician is convinced that a patient should not be treated (for whatever reason), the patient should **NOT **be included in the trial. Patients should only be randomised if they fulfil the eligibility criteria **AND **the clinician is substantially uncertain about the balance of risks and benefits of rt-PA for that individual.

### Consent

IST-3 will be run according to the standards laid out in the MRC Guidelines for Good Clinical Practice in Clinical Trials (United Kingdom) and in keeping with the EU directive on Clinical Trials. These guidelines are based on the ICH Harmonised Tripartite Guideline for Good Clinical Practice and the Declaration of Helsinki. Local Ethics Committee (or local equivalent) approval is needed for each participating centre before recruitment can begin. The consent process was developed, in line with recent recommendations[28], with consumer involvement[29]. Consent is supported by a patient (or carer) information leaflet (Appendices 2 and 3) and is adapted to local ethical requirements and the clinical state of the patient:

• If patients can understand and write, signed consent must be obtained.

• Patients who can comprehend, but are unable to write, may provide verbal witnessed consent.

• The patient's relative or spouse may act as the patient's personal legal representative and provide assent to trial inclusion (consent) if the patient is acutely mentally incompetent as a result of their stroke (e.g. aphasia or decreased conscious level).

• Under certain strict criteria, if no relative is available, some local ethics committees may permit a professional legal representative, such as an independent doctor, to enable those patients unable to give consent to be recruited (this is acceptable in certain emergency situations and sometimes previously called 'a waiver of consent')[28].

• The requirements of the relevant ethics committee should be adhered to at all times.

### Brain imaging

All patients MUST have a pre-randomisation brain scan to exclude intracranial haemorrhage. CT scans should cover the entire brain from the foramen magnum to the vertex with 4 – 5 mm thick slices through the posterior fossa and 8 – 10 mm thick for the cerebral hemispheres, with no slice gap. Scans should be windowed on a width of 80 Hounsfield Units (HU) and a centre level of 35 – 40 HU. This is particularly important if scans are to be sent as printed film. All patients (irrespective of treatment allocation) MUST have a follow-up scan at 24–48 hours. In addition a repeat scan is required if the patient deteriorates neurologically or intracranial haemorrhage is suspected for any reason. Although CT scanning is preferred, MR brain imaging is allowed provided there is sufficient radiological support in the hospital to interpret the scans and a gradient echo (T_2 _*) is included to exclude haemorrhage (haemorrhage can be overlooked on several other types of MR imaging sequence) and Diffusion Weighted Imaging (DWI) is required to identify the recent infarct. All scans performed during the first 7 days following randomisation are to be sent to Edinburgh for coding. The two sets of CT scans per patient (more, if the patient had extra scans due to suspected complications) are to be sent to the Edinburgh trials office, either by post, or (subject to certain conditions) by electronic transfer of DICOM files (details of methods of file transfer and copies of the Scan transfer forms are given in the trial operations manual). If sending a hard copy film, the original is to be sent, as this allows better conversion to an electronic file (a copy should be made and kept at the treating hospital). Hard copy scans will be digitised and converted to DICOM files. All images will be coded with all original identifiers stripped from the record. Each scan can then be assessed, blind to patient details, and to whether the scan is pre- or post treatment. Each scan will be assessed by an international panel of expert radiologists by means of an internet web-based computer system.

### Advanced imaging substudies

IST-3 will permit advanced imaging substudies in centres with appropriate facilities and local expertise. Such studies could include CT angiography, MR diffusion and perfusion imaging, carotid duplex and transcranial doppler imaging. Any such proposed sub-studies must be approved by the IST-3 Trial Steering Committee.

### Randomisation

The clinician enters patients by telephone call to an automated randomisation system available 24 hours a day. The randomisation system requests a few key items of baseline data, which are then entered with the telephone keypad. A web-based randomisation is being planned for 2006. When the data have been entered and checked, the computer generates the treatment allocation. The system includes a standard minimisation algorithm which ensures that the treatment groups are balanced for key prognostic factors[30]. The algorithm balances allocation on stroke severity (calculated as the patient's predicted probability of a poor outcome, calculated from a validated prognostic model based on key clinical variables measured at baseline)[31]. Patients allocated 'immediate rt-PA' should be treated as soon as possible after the randomisation call is completed.

### Trial infusions

All patients should have intravenous access in place and be administered intravenous fluid therapy according to local acute care protocols. Patients allocated 'immediate rt-PA' should be given recombinant tissue-type plasminogen activator (Alteplase, Boehringer Ingelheim; or Activase, Genentech) in a total dose of 0.9 mg per kg of body weight up to a maximum of 90 mg. Ten per cent of the dose is given as an intravenous bolus delivered over one minute followed by the rest of the infusion over the next 60 minutes. Patients allocated 'control' must avoid treatment with rt-PA and should receive stroke care in the same clinical environment as those allocated 'immediate rt-PA'. Both treatment groups must have their blood pressure monitored closely over the first 24 hours, according to the IST-3 protocol, and this must be documented. Both groups should receive the same general supportive care.

### Management protocols

All patients entered in the trial, whether allocated rt-PA or control, must be managed according to local acute stroke care protocols, in the same clinical environment. Such protocols are not specified by the trial, but will generally include the components of effective stroke unit care[25]. Soon after admission, intravenous access, monitoring of physiological variables, correction of any abnormalities, and where clinically appropriate, intravenous fluid therapy should be initiated.

### Blood pressure: monitoring and intervention

The NINDS group specified a detailed protocol for the active lowering of blood pressure, though it was unclear whether this policy was beneficial or harmful to patients in the trial[32]. The Blood Pressure in Acute Stroke Collaboration (BASC), have since reviewed all the relevant randomised controlled trials of blood pressure lowering in acute stroke[33] and concluded (as did the International Society for Hypertension [ISH] [34] that there were no data from reliable randomised controlled trials to guide the management of high blood pressure in patients with acute stroke. Blood pressure tends to fall in the acute phase of stroke and in view of the conclusions of the BASC and ISH, no particular IST-3 protocol for blood pressure management will be specified. To monitor any interaction between blood pressure and response to treatment in IST-3, data on blood pressure levels and the use of blood pressure lowering treatments will be collected. This aspect of the trial will be monitored by the Data Monitoring Committee.

### Symptomatic intracranial bleeding

Intracranial haemorrhage should be suspected if any of the following occur during the infusion or within 24 hours of randomisation:

• Neurological deterioration.

• New headache.

• New acute hypertension.

• New nausea or vomiting.

• Sudden decrease in conscious level.

If any of these events occur, any rt-PA infusion should be stopped and the patient examined for possible reasons for the deterioration. Blood should be taken to measure prothrombin time (PT), activated partial thromboplastin time (APPT), fibrinogen, full blood count and group and save serum. CT scanning must be performed immediately, irrespective of the allocated treatment group. If CT scanning confirms intracranial haemorrhage, rt-PA must not be restarted. Management should follow local protocols and will usually require consultation with a haematologist and a neurosurgeon. For patients who have received rt-PA there is no reliable evidence available to recommend any one treatment strategy over another, but fibrinolytic inhibitors such as tranexamic acid may be useful. In the rare instance that fibrinogen levels are low (<1 g/L) after rt-PA therapy, cryoprecipitate (containing fibrinogen and factor VIII) may be required[35]. Fibrinogen assays vary but the Clauss technique is considered the best method if available[36].

### Asymptomatic intracranial bleeding

If asymptomatic intracranial bleeding (haemorrhagic transformation of the infarct or parenchymatous haematoma) is detected on the repeat CT scan performed at about 24 hours after randomisation, no specific action is needed, but it may be necessary to delay the start of long-term antiplatelet or anticoagulant therapy. The degree of delay is a matter for the responsible clinician to determine, but will be influenced by factors such as the degree and extent of haemorrhage, the patient's clinical condition, the nature of the planned treatment and the indication for its use.

### Extra-cranial bleeding

If significant extra-cranial bleeding develops, any rt-PA infusion must be stopped immediately. Blood should be taken to assess prothrombin time (PT), activated partial thromboplastin time (APPT), fibrinogen, full blood count and cross match. Appropriate supportive therapy should be given, with monitoring of blood pressure, maintenance of circulating blood volume with intravenous fluids and transfusion of blood as appropriate. The results of the investigations will guide emergency treatment. Management should follow local protocols and will usually require consultation with a haematologist. For patients who have received rt-PA there is no reliable evidence available to recommend any one treatment strategy over another, but fibrinolytic inhibitors such as tranexamic acid may be useful. If fibrinogen levels are low (<1 g/L) cryoprecipitate (containing fibrinogen and factor VIII) may be required[35]. Fibrinogen assays vary but the Clauss technique is considered the best method if available[36].

### Allergic or hypersensitivity reactions

Anaphylactic reactions can occur following rt-PA administration for acute ischaemic stroke, but occur rarely[37]. If there are any signs of anaphylactic response or hypersensitivity (e.g. peri-orbital swelling, tongue swelling, urticarial rash) any rt-PA infusion should be stopped immediately. Patients require urgent medical assessment ('airway, breathing and circulation'). Treatment will depend on the severity of the reaction. Intravenous steroids and antihistamines may be sufficient for mild reactions. Adrenaline (nebulised or intramuscular) and intubation may be required for severe reactions. Local advice from the emergency medicine team should be sought. All such reactions should be recorded on the 7-day hospital form.

### Other aspects of treatment

#### Antithrombotic treatment should not be given within the first 24 hours of the start of rt-PA treatment

There is some evidence to suggest that early aspirin, given with thrombolytic therapy, may increase the risk of death[38]. Antithrombotic treatment (antiplatelet drugs and heparin) should therefore be avoided in the first 24 hours after start of trial treatment for patients who have received rt-PA. Patients treated with rt-PA should first have a repeat CT brain scan, performed at 24–48 hours after treatment, and start long-term antiplatelet therapy with aspirin or other agents, only if the second CT has excluded intracranial haemorrhage. Patients allocated control should start long-term antiplatelet therapy with aspirin (or other effective antiplatelet agent) after randomisation according to usual practice. There are no data to suggest that this delay in initiating antiplatelet drugs materially disadvantages rt-PA allocated patients. The modest benefit of aspirin, given at 24–48 hours after onset of stroke symptoms, was similar to that when given within the first few hours[3]. Conversely, the earlier use of aspirin for patients allocated control is therefore unlikely to introduce a major difference between rt-PA and control groups and will anyway reflect usual clinical practice for control patients. All antithrombotic medication used in the first week following treatment will be recorded on the 7 day trial form to monitor deviation from the protocol and assess risk factors for side effects.

#### Long-term antiplatelet drugs

Unless there is a clear contraindication, all patients should be considered for long-term antiplatelet therapy with aspirin (or other effective antiplatelet) for routine secondary prevention of vascular events[39]. Treatment should not be started until 24 hours after any rt-PA infusion (see above). At discharge, all patients will be given a card recording their participation in the study and their General Practitioners should be informed by letter.

#### Other treatments in hospital

All other aspects of treatment are at the discretion of the responsible clinician.

### Follow-up

All patients will be followed up, whether they complied with their treatment or not. Follow-up will be at seven days, hospital discharge, transfer to another hospital or death, whichever occurs first. The Hospital Co-ordinator at each collaborating centre must complete the hospital follow-up form for each patient, and send it to the IST-3 Trial Office, enclosing a copy of the pre-and all post-randomisation CT scans.

Six months after randomisation, General Practitioners (or Hospital Co-ordinators) will be contacted by the IST-3 Trial Office staff to check that their patient is alive and that they may be approached for follow-up. If appropriate, the IST-3 Trial Office staff will then mail a postal questionnaire to patients, to record dependency and health related quality of life. The exact procedures for follow-up in each country will be decided by the National Co-ordinator and the IST-3 Management committee. Central follow-up (telephone or postal) has been found to be cost-effective, efficient and also ensures blinding of the assessment. If a patient dies after a hospital follow-up form has been completed (up to 7 days from randomisation), and within 6 months of randomisation, the clinician can conveniently inform the IST-3 Trial Office by completing and returning a simple form to reduce the risk of the co-ordinating centre mailing a questionnaire to a patient who has died. The precise date of death will be very important for survival analyses.

To assess the durability of any treatment benefit beyond 6 months, patients recruited in the UK (and in other countries where appropriate funding has been obtained) will be followed up one year after the six month assessment and annually thereafter (dependent on sufficient funding). These data will also permit more detailed health economic modelling and to test the hypothesis, that the level of disability at six months predicts long-term survival.

Patients may withdraw consent to participate in the trial at any stage. This may involve withdrawal from trial treatment (in which case any rt-PA infusion should be stopped) or withdrawal from trial follow-up. If the latter, it is essential to obtain their consent at the point of withdrawal to obtain follow-up information on their outcome from other sources, e.g. from their hospital records, their general practitioner or central health services data.

## Outcome events

### Primary

The primary measure of outcome is the proportion of patients alive and independent (i.e. Modified Rankin Score 0–2) assessed by validated postal/telephone questionnaires six months after randomisation[40,41].

### Secondary

#### a) Fatal events within 7 days

• **Deaths from any cause**

• **Deaths within 7 days, subdivided by cause of death**. Deaths attributed to neurological causes will be categorised as follows: death due to swelling of the initial infarct; death due to intracranial haemorrhage; death due to the initial stroke, but not attributable to infarct swelling or haemorrhage; death due to recurrent ischaemic stroke; or death due to recurrent stroke of unknown type.

#### b) Non-fatal events within 7 days. The occurrence of one of the following events within 7 days, in a patient alive at 7 days

• **Symptomatic intracranial haemorrhage**. In a patient with ***either ***a clinically important worsening of their deficit measured on a valid stroke scale, ***or ***the occurrence of a clinical syndrome suggesting recurrent stroke, the presence of significant intracranial haemorrhage on a CT or MR scan performed within 7 days of randomisation.

• **Recurrent ischaemic stroke**. Further stroke in a different vascular territory to the index stroke, according to clinical features. The diagnosis must be supported by brain imaging to exclude haemorrhage (but not necessarily to confirm the vascular territory of the new infarct).

• **Recurrent stroke of unknown type**. Further stroke in a different vascular territory to the index stroke, according to clinical features, but with no brain imaging or autopsy performed.

• **Neurological deterioration attributed to swelling of the initial ischaemic stroke**. In a patient with relevant clinical deterioration, the presence of significant cerebral oedema (i.e. complete ventricular effacement, midline shift or obliteration of the basal cisterns) on a post-randomisation CT scan (or MR) performed within 7 days of randomisation.

• **Neurological deterioration not attributable to swelling of the initial ischaemic stroke or haemorrhage**. A patient with relevant clinical deterioration, but no evidence on CT or MR of significant swelling or haemorrhage.

#### c) Other events within 7 days

• **Major extracranial haemorrhage **(i.e. fatal, severe enough to require transfusion or operation, or an absolute decrease in haemoglobin ≥ 5 g/dL or a decrease in haematocrit of ≥ 15% or bleeding associated with persistent or serious disability).

• **Asymptomatic intracranial haemorrhage identified by routine repeat brain imaging**. The presence of any intracranial haemorrhage on a CT scan (or MR scan) performed within 7 days of randomisation with no clinical deterioration (i.e. no corresponding worsening of neurological deficit, and no evidence of recurrent stroke)

#### d) Outcome at six months (and, for UK patients, at 18 months and annually thereafter)

• Number of patients dead from any cause within six months

• Number of patients dead from a vascular cause (includes death due to bleeding) within six months

• Number of patients making a complete recovery from the stroke (defined by simple recovery question)[41]

• Oxford Handicap Score (Modified Rankin)[40]

• 'Dependency' question[41]

• EQ-5D (EuroQol) questionnaire[42,43].

• Residence at six months (i.e. at home, still in hospital, long-term geriatric ward, nursing home, residential home, with relatives or other)

## Analyses

'Intention-to-treat' analyses will be used throughout.

### Primary analysis

The primary analysis will be a comparison of the proportion of patients in each group who are alive and independent at six months (Modified Rankin 0,1 and 2), for all those allocated rt-PA versus all those allocated control.

### Pre-planned subgroup analyses

Analyses will be performed of the effect of treatment at six months among all those allocated rt-PA versus all those allocated control, subdivided by the following baseline features:

• Time from onset to randomisation (0–3 vs. 3–6 hours)

• Age

• Gender

• Clinical stroke syndrome using the OCSP classification[44]

• Presence or absence of atrial fibrillation (AF)

• Pre-randomisation brain imaging appearances (extent of visible infarction, visible infarct versus not, small vessel disease and atrophy)

a) as assessed by the randomising clinician (recorded at baseline), and

b) as assessed by independent blinded review of the pre-randomisation scan

• Use of antiplatelet drug treatment at the time of randomisation

• Stroke severity according to the NIHSS.

• Blood pressure at randomisation

• Randomisation in a centre with prior experience (treatment of more than 3 patients with thrombolysis in the 12 months prior to the start of the trial) versus not

• Randomisation during the double blind start-up phase vs. randomisation during the main (open) phase.

A variety of other subsidiary analyses, will be performed of the effect of treatment on: death from any cause within the first seven days; death from any cause at six months; death from vascular causes at six months; fatal intracranial haemorrhage; complete recovery at six months; outcome as measured by the Oxford Handicap Score[40]. Treatment effects on the primary outcome and secondary outcomes, subdivided by other baseline features will be performed as appropriate, with due allowance for their exploratory nature.

### Collaboration with systematic reviews of thrombolysis

When the main report of IST-3 has been published, the IST-3 group will collaborate with the authors of the Cochrane systematic review and with the rt-PA Study Group to include the trial data from the trial in their analyses.

### Sample size

The IST-3 has a planned sample size of 6,000 patients. Assuming a power of 80%, an alpha level of 5%, and the same control event rate as observed in the trial to date (confidential data), with 6000 patients, mostly treated between 3 & 6 hours of onset, the trial could detect a 3% absolute difference in the primary outcome (the proportion of patients dead or dependent at 6 months). This absolute difference is clinically worthwhile, is consistent with the effect size observed among patients randomised between 3 & 6 hours of stroke onset in the Cochrane review of the rt-PA trials. It is also comparable with the absolute benefit seen with thrombolytic therapy for acute MI. If 3500 patients were recruited, the trial could detect a 4% absolute difference in the primary outcome. A sample size of 1000 patients could detect a 7% absolute difference in the primary outcome, which is consistent with the effect size among patients randomised within 3 hours of stroke in the Cochrane review.

## Data and safety monitoring

### Interim analyses: role of the Data Monitoring Committee

During the period of recruitment into the study, interim analyses of the proportion of patients alive and independent and the numbers of total deaths at six months and analyses of other major outcome events will be supplied, in strict confidence, to the chairman of the data monitoring committee, along with any other analyses that the committee may request. In the light of these analyses, the data monitoring committee will advise the chairman of the steering committee if, in their view, the randomised comparisons have provided both (i) 'proof beyond reasonable doubt' that for all, or some, the treatment is clearly indicated or clearly contra-indicated and (ii) evidence that might reasonably be expected to materially influence future patient management. Appropriate criteria of proof beyond reasonable doubt cannot be specified precisely, but the DMC will work on the principle that a difference of at least 3 standard errors in an interim analysis of a major outcome event (e.g. death from all causes or independent survival at six months) may be needed to justify halting, or modifying, a study before the planned completed recruitment. This criterion has the practical advantage that the exact number of interim analyses would be of little importance, and so no fixed schedule is proposed[45]. Following a report from the DMC, the steering committee will decide whether to modify entry to the study (or seek extra data). Unless this happens however, the steering committee, the collaborators and central administrative staff will remain ignorant of the interim results.

### Monitoring of Suspected Unexpected Serious Adverse Reactions to the study treatment (SUSARs)

Throughout the trial, all suspected unexpected serious adverse reactions believed with reasonable probability to be due to study treatment are to be reported immediately by telephoning the 24-hour telephone service (the randomisation 'helpline' +44 (0)131 537 2953). During this telephone call, an initial SUSAR report will be completed using information provided by the person reporting the event. The information recorded will provide standard preliminary details of the event (i.e. identity of the patient and of the person reporting the event, nature and date of the event, and reasons for attribution to the study treatment etc). These reports will be reviewed within 48 hours by the study clinical co-ordinators. Any additional important further information will be sought urgently. If the event is assessed as being 'expected', the decision will be recorded on the SUSAR form and a copy of the outcome sent to the reporting centre. If the event is confirmed as 'unexpected' and classified as fatal or life threatening, a full SUSAR report of the event will be requested. This report will be sent, within 7 days to: the Chairman of the Data Monitoring Committee, the Chairman of the Multi-centre Research Ethics Committee, the Medicines and Healthcare Products Regulatory Authority, The department for Research and Development (Edinburgh) and all Principal Investigators involved in the trial. SUSARs, which are not classified as fatal or life threatening will be reported within 15 days to all persons and agencies as specified above.

Serious adverse reactions are defined as those which are either life threatening, require in-patient hospitalisation or prolongation of existing hospitalisation, result in persistent or significant disability or incapacity, are congenital anomalies or birth defects. 'Unexpected' means not included in the list of known adverse drug reactions recorded in the summary of product characteristics (listed below).

### Expected adverse events

The most frequent adverse reaction associated with rt-PA is bleeding resulting in a fall in haematocrit and/or haemoglobin values. The type of bleeds associated with thrombolytic therapy can be divided into two broad categories: superficial bleeding, normally from arterial or venous puncture sites or damaged blood vessels; internal bleeding into the gastro-intestinal or uro-genital tract, retro-peritoneum or central nervous system or bleeding of parenchymatous organs. Symptomatic intracerebral haemorrhage is the main adverse event of Actilyse in treatment of acute ischaemic stroke (up to 10% of patients). In clinical studies with Actilyse, significant blood-loss was observed occasionally from gastro-intestinal bleeding, uro-genital or retro-peritoneal bleeding. Ecchymosis, epistaxis and gingival bleeding are observed rather frequently but usually do not require any specific action. Actilyse therapy may lead to cholesterol crystal embolisation or thrombotic embolisation in rare cases. In the organs concerned, this may lead to corresponding consequences (e.g. renal failure in the case of renal involvement). In patients receiving Actilyse for myocardial infarction successful reperfusion is often accompanied by arrhythmias. These may require the use of conventional antiarrhythmic therapies. Patients with myocardial infarction or pulmonary embolism may experience disease-related events such as cardiac failure, recurrent ischaemia, angina, cardiac arrest, cardiogenic shock, reinfarction, valve disorders (e.g. aortic valve rupture), and pulmonary embolism. These events have also been reported following thrombolytic therapy and can be life-threatening and may lead to death. In rare cases nausea, vomiting, drop in blood pressure and increased temperature have been reported. These reactions can also occur as concomitant symptoms of myocardial infarction. As with other thrombolytic agents, events related to the central nervous system (e.g. convulsions) have been reported in isolated cases, often in association with concurrent ischaemic or haemorrhagic cerebrovascular events. In rare cases, anaphylactoid reactions have been reported. These are usually mild, but can be life-threatening in isolated cases. They may appear as rash, urticaria, bronchospasm, angio-oedema, hypotension, shock or any other symptom associated with allergic reactions. If they occur, conventional anti-allergic therapy should be initiated. Transient antibody formation to Actilyse has been observed in rare cases and with low titres, but a clinical relevance of this finding could not be established.

### Compliance with Good Clinical Practice Guidelines

The trial will conform to the MRC Guidelines for Good Clinical Practice in Clinical Trials[46]. Trial data will be checked for validity and internal consistency and various measures will be taken to identify any scientific misconduct (details of such measures remain confidential for obvious reasons). Centre visits will be carried out and primary records inspected.

## Assessment and storage of brain images

### Collection and storage

CT and MR brain scans at baseline and follow-up are to be sent by secure mail or electronic means to Edinburgh. Anonymised digital copies of these scans will be stored on computer servers for analysis and archiving. The systems have been designed to ensure the highest levels of data security and patient confidentiality, and will be further enhanced if future technological advances permit it. The enhancements to the current system may include the use of e-Science and Grid technologies if they prove to be superior to current systems. The use of e-Science infrastructure within the MRC Neurogrid project for the IST-3 imaging data could: ensure more reliable, secure and confidential archiving of the imaging data; connect sites for rapid and secure flow of data; enable distributed data analysis with image analysis tools; enhance collaborative working between members of the research team; and, improve the power and applicability of studies.

### Assessment

All brain scans (baseline and follow-up) are to be assessed by at least one expert reader, by means of a web-based image assessment tool which presents anonymised images to the reader. The image data remain on the trial server; the system presents anonymous images in Joint Photographic Experts Group (JPEG) format (with no personal or demographic or other information) together with a structured questionnaire on the same screen to the assessor. The assessor then records their interpretation of the scan by means of the on-screen questionnaire. The scan interpretation is then stored directly on the secure IST3 trial database, with no need for email, fax or postal transmission of data. The Image Reading Advisory panel will advise on the conduct of this work, on the size of the CT reading panel, and on the selection of readers.

## Roles and responsibilities

### Role of the Steering Committee

The Committee will be responsible for overseeing the conduct of the trial. It shall be constituted and operate as laid out in the MRC Guidelines for Good Clinical Practice[46].

### Role of the International Advisory Board

This will constitute the National Co-ordinators from each participating country, representatives of other major trials and other individuals with relevant expertise who may be co-opted as appropriate. The Board will be chaired by Chairman of the Trial Management Group. The Board fulfils two roles: a) to advise the trial management team and the trial steering committee on matters relevant to the trial, and b) to enable appropriate representation of the National Co-ordinators views on the trial. Advice from the Board to the steering committee and management committee is not binding.

### Role of the Management Group

The group is responsible for all aspects of day to day management of the trial and is based at the Neurosciences Trials Unit at Edinburgh University. It is responsible for: the recruitment of trial centres; provision of training material for the collaborating centres; organising trial meetings and training meetings; provision of trial materials; data collection, checking and data entering; trial analysis; co-ordinating the production of trial reports and publications.

### Responsibilities of the National Co-ordinators

National Co-ordinators will represent the IST-3 in their country. The National co-ordinator should: approve new local co-ordinators before they submit any local ethics committee application; undertake the centralised follow-up at six months in their country; liaise with the IST-3 Management Group over the conduct of national follow-up procedures; help maintain a high profile for IST-3 in their country and encourage appropriate recruitment; attend meetings of the International Advisory Board to represent the views of participants in their country.

### Responsibility of Local Co-ordinators

Local co-ordinators will represent the IST-3 in their centre (hospital). It is expected that local co-ordinators work in a well organised stroke service, preferably including a stroke unit. The local co-ordinator should: liaise with the National Co-ordinator prior to any local ethics application and trial start-up in their hospital; maintain a high standard of stroke assessment and 'fast-tracking' of potential participants in their hospital, supported by a written protocol; liaise with local neuroradiology or radiology colleagues to ensure immediate access to CT brain imaging; liaise with appropriate emergency medicine colleagues; be responsible for continuous medical education to maintain appropriate high standards of care for patients considered and randomised in the trial (this will usually involve regular meetings with medical, nursing, allied health care staff in the emergency department and stroke unit); ensure compliance with Good Clinical Practice Guidelines.

### Role of the independent events adjudicator

An expert independent clinician will review – blinded to treatment allocation -clinical and radiological information on any significant cerebral event that occurs up to 7 days after randomisation. The classification of such events will be compared with that assigned by two senior members of the trial management staff and any differences will be resolved by discussion. The agreed assessment of each event will form part of the data reviewed in confidence by the Data Monitoring Committee.

### Role of the image reading advisory group

All brain scans (baseline and follow-up), will be reviewed by a panel of expert readers. The methods to be used by the panel of readers, the overall conduct of the image reading process and the interpretation of the findings at the end of the study will be guided by the image reading advisory group. Suitably qualified experts will be invited to join the group which is chaired by Professor Wardlaw.

### Non-negligent liability

There are no special arrangements for non-negligent liability in the IST-3. Patients will be protected by the usual arrangements for negligence in the participating hospitals.

### Publication in the names of all collaborators

The success of this study depends entirely on the collaboration of a large number of doctors, nurse and patients. For this reason the credit for the main results will be given, not to the central trial co-ordinators, but to all wholehearted collaborators in the study. The primary trial publication will be drafted by a writing committee whose membership has been approved by the steering committee. The manuscript must be approved by the steering committee before submission for publication.

### Non-trial thrombolysis

The IST-3 Group recommends that any non-trial thrombolysis treatment for stroke is registered in post-marketing studies such as SITS-MOST[47].

## Trial organisation

### Co-ordinating centre (for all information and queries)

IST-3 Co-ordinating Centre[48], Department of Clinical Neurosciences, University of Edinburgh, Bramwell Dott Building, Western General Hospital, Edinburgh, UK, EH4 2XU. email: ist3@skull.dcn.ed.ac.uk, telephone: +44 (0)131 537 2793 fax: + 44 (0)131 332 5150.

### Steering committee

Independent Chairman: Professor David Chadwick, University of Liverpool, (2000–2008); Professor Colin Baigent, University of Oxford, (2008-); Independent members: Dr Pippa Tyrrell (Manchester University), Professor Gordon Lowe (Glasgow University); Co-principal Investigators: Professor Peter Sandercock (Edinburgh University); Professor Richard Lindley (Professor of Geriatric Medicine, University of Sydney); Statistician: Dr Stephanie Lewis; Neuroradiology Advisor: Professor Joanna Wardlaw (Edinburgh University); Trial Manager: Ms Karen Innes.

### IST-3 management group

Professor Peter Sandercock (Chairman and co-ordinator for trial centres in Europe and North America), Ms Karen Innes (Trial Manager), Professor Joanna Wardlaw (Neuroradiology), Professor Martin Dennis (Stroke Services). Dr Stephanie Lewis (Trial Statistician), Professor Richard Lindley (Co-ordinator for centres in Australasia)

### International Advisory Board

National Co-ordinator from each country, Professor Charles Warlow (Neurology and Clinical Trials), Professor Gary Ford (UK Thrombolysis Advisor), Dr Markku Kaste (ECASS-3 trial liaison), Dr John Marler (NINDS Liaison), Professor Stephen Davis (EPITHET trial liaison).

### Independent Data Monitoring Committee (DMC)

Professor Rory Collins, Oxford University, UK (Chairman), Professor Philip Bath (Nottingham University), Professor Jan van Gijn (University of Utrecht, The Netherlands) (2000–2008), Professor Richard Gray (University of Birmingham), Dr Salim Yusuf (McMaster University, Canada), Professor Robert Hart (University of San Antonio) (2008-).

### Independent events adjudicator

Dr Keith Muir, Institute of Neurological Sciences, Glasgow.

### Sponsorship

The University of Edinburgh and the Lothian Health Board act as joint sponsors for the study and hold the Clinical Trial Authorisation.

## List of abbreviations

APPT: Activated Partial Thromboplastin Time; ATLANTIS: Alteplase Thrombolysis for Acute Noninterventional Therapy in Ischemic Stroke; BASC: Blood Pressure in Acute Stroke Collaboration; BP: Blood Pressure; CT: Computed Tomography; DICOM: Digital Imaging and Communications in Medicine Standard; DMC: Data Monitoring Committee; DWI: Diffusion Weighted Imaging; ECASS: European Cooperative Acute Stroke Study; EPITHET: Echoplanar Imaging Thrombolytic Evaluation Trial; HU: Hounsfield Units; ICH: International Committee for Harmonisation; ISH: International Society for Hypertension; IST-3: Third International Stroke Trial; JPEG: Joint Photographic Experts Group; MR: Magnetic Resonance; MRC: Medical Research Council; MRI: Magnetic Resonance Imaging; NINDS: National Institute of Neurological Disorders and Stroke; PT: Prothrombin Time; OR: Odds Ratio; PROBE: Prospective Randomised Open Blinded Endpoint Design; rt-PA: Recombinant Tissue Plasminogen Activator; SUSAR: Suspected Unexpected Serious Adverse Reaction; TSC: Trial Steering Committee

## Competing interests

Peter Sandercock:

1. Was the Principal Investigator of the second International Stroke Trial (IST-2) to evaluate a neuroprotective compound (619c89). The trial was to be conducted independently of the manufacturer (Glaxo-Wellcome). Peter Sandercock received an initial grant of £800,000 from Glaxo-Wellcome for the initial phases of the study, but the compound was withdrawn from clinical development and IST-2 was terminated before any patients had been randomised.

2. Has received lecture fees and travel expenses from Bayer and from Boehringer Ingelheim for lectures given at international conferences.

3. He serves on the Independent Data Monitoring and Safety Board of the RELY trial, funded by Boehringer Ingelheim and receives attendance fees and travel expenses for attending board meetings.

4. He does not have any paid consultancies with pharmaceutical companies, and is not a member of the Speaker's Panel of any company.

Karsten Bruins Slot:

• Received an honorarium for a lecture from Boehringer Ingelheim and had costs for participating in scientific meetings reimbursed.

Gord Gubitz:

• Received honoraria and speaker fees from: Boehringer Ingelheim, Sanofi Synthlabo Aventis, Hoffman La Roche and Novo Nordisk.

Veronica Murray:

• Received an unrestricted educational grant for a meeting on thrombolysis in stroke at which IST-3 was discussed.

## Authors' contributions

The study was conceived by the co-chief investigators, PS and RL. JW led the development of all of the imaging aspects of the study. The study was designed by the co-chief investigators, MD and SL, with input from all the other listed contributors who act as coordinators of the trial in their own country. All authors have read and approved the manuscript.

## Supplementary Material

Additional file 1 Board Assessment template. Medical Research Council review of trial. Click here for file

Additional file 2 Multicentre Research Ethics approval. Multicentre Research Ethics approval. Click here for file

Additional file 3 Multicentre Research Ethics approval. Multicentre Research Ethics approval. Click here for file

Additional file 4 Multicentre Research Ethics approval of amendment. Multicentre Research Ethics approval. Click here for file
