# Supplementary material for: The third international stroke trial (IST-3) of thrombolysis for acute ischaemic stroke
Source: Trials. 2008 Jun 17;9:37. doi: 10.1186/1745-6215-9-37 (PMC2442584; doi:10.1186/1745-6215-9-37)
Supplement: Additional file 1 — Board Assessment template. Medical Research Council review of trial. [file 1745-6215-9-37-S1.pdf]

Board Assessment Template

IN CONFIDENCE

Medical Research Council	Health Services and Public Health Research Board (HSPHRB)	14/11/2007
	File reference:	G0400069
	Agreement ID:	85116
	Minute:	12(ii)

BACKGROUND INFORMATION

PRINCIPAL INVESTIGATOR:	Professor P A G Sandercock et al
DEPARTMENT/LOCATION:	University of Edinburgh, University of Edinburgh
TITLE OF GRANT/NAME OF UNIT:	Third International Stroke Trial: IST-3
FORM OF SUPPORT:	Trial Grant – request for extension and supplementation
START DATE & DURATION:	01/04/2010 for 24 months

BOARD SCORE:	4.5	TOTAL MRC CONTRIBUTION REQUESTED(£K, INDEXED):	277 (additional to costs already awarded)
		DECISION:	**AWARD**

ABSTRACT

IST-3 is an international, multi-centre, randomised, controlled trial of iv rt-PA (0.9 mg/kg) within 6 hours of onset of acute ischaemic stroke. All potential patients must be assessed and have brain imaging without delay. CT scanning is the prime imaging modality, but CT perfusion/ CT angiography, MRI DWI/PWI/angiography and transcranial doppler can be used as additional selection criteria in centres which participate in the IST3 imaging substudy. Patients who meet clinical and brain imaging criteria are entered by means of a telephone call to a fast centralised computer system which records baseline data, balances on key prognostic factors and allocates treatment. Follow-up is conducted by postal or telephone questionnaire, independently of the clinician treating the patient. The primary measure of outcome is the proportion of patients who are dead or dependent at six months (poor functional outcome). The trial involves innovative methods to make a large scale trial feasible. The web-based CT reading tool developed in the start-up phase is already being applied in several other imaging research projects. The trial builds on our NHS HTA formal economic modelling exercise and will provide reliable evidence on the place of this controversial treatment in routine clinical practice in the NHS (and other publicly funded Health Services). The trial will also provide important - and much needed - randomised trial data on the balance of risk and benefit of this treatment in older people, who have been deliberately excluded from earlier trials of thrombolysis.

BOARD ASSESSMENT

Assessment of the Proposed Research

The Board noted that MRC funding for the main phase of the IST-3 trial was assessed by HSPHRB in June 2004. The Board recognised the importance of this trial particularly in relation to older patients and high-risk groups and the application was recommended for funding.The IST-3 trial was aimed to establish evidence on the use of thrombolysis in patients within 3h and for use beyond the current licence a) between 3h and 6h after onset and b) in patients over 80 years of age who suffer 33% of total strokes.

The Board considered the three options presented for the extension of the IST-3 study, with closeout by 2010, 2011 and 2012 respectively. Correspondingly, the overall target for recruitment would be lowered from 6000 to 1866, 2483 and 3100 patients. Members noted the letter from the Trial Steering Committee that gave strong endorsement to the third scenario, recruitment until July 2011 followed by closeout in April 2012. The continuing importance of the trial was confirmed to the MRC by the Stroke Association.

Much of the discussion focused on the potential for future recruitment, given the difficulties to date which had required time and effort to overcome. Issues of governance appeared now however to be mostly resolved. The applicants had put the case that recruitment would soon become easier because the new national strategies for stroke would require equitable access to thrombolysis across the UK and this in turn would help the stroke research networks to become more effective in encouraging recruitment of patients into IST-3.

The Board accepted that the trial would add little to the literature if it continued as originally planned. Members took the view that the justification for extending the trial rested on the ability to provide new information on the benefits or risks to patients currently excluded from thrombolysis treatment. In addition to providing new information, extension of the trial under option 3 would allow ‘death from all causes’ to be added as an end point to the systematic review of thrombolysis treatment for acute stroke and thus contribute to future metaanalysis. More broadly, the availability of effective treatment for stroke in the UK compared unfavourably with treatment for myocardial infarction. The trial offered probably the last opportunity to provide an evidence base for thrombolyis in acute stroke.

The proposals were from a strong well-established group with considerable experience in running clinical trials. The Board noted that continued efforts to enhance recruitment would be required for the trial to succeed in meeting the new targets. Given the expected outcomes and the level of resources requested, the proposal for a two year extension was agreed to offer the best value for money. Savings from the early months of the trial had already been factored into the extension request.

In conclusion the Board agreed that the scientific question remained valid and there was a good case to fund a two year extension to the IST-3 trial, which was in the hands of an experienced and committed team.

The Board did not identify any new ethical issues in the request for extension.

Level of Support Recommended

Detail how the resources recommended for award differ from those requested in the proposal, providing an explicit list of ***all prunings*** *recommended by the Board.*

N/A
