# Supplementary material for: The third international stroke trial (IST-3) of thrombolysis for acute ischaemic stroke
Source: Trials. 2008 Jun 17;9:37. doi: 10.1186/1745-6215-9-37 (PMC2442584; doi:10.1186/1745-6215-9-37)
Supplement: Additional file 2 — Multicentre Research Ethics approval. Multicentre Research Ethics approval. [file 1745-6215-9-37-S2.pdf]

MULTI-CENTRE RESEARCH ETHICS COMMITTEE FOR SCOTLAND

Deaconess House
148 Pleasance
Edinburgh EH8 9RS

Telephone 0131-536-9026
Fax 0131-536-9346
E-mail: walter.hunter@lhb.scot.nhs.uk
Website: http://dspace.dial.pipex.com/mrec

Reference: WH/MREC/99/0/78

Dr Richard Lindley
Department of Clinical Neurosciences
Bramwell Dott Building
Western General Hospital
Edinburgh
EH4 2XU

12 November 1999

Dear Dr Lindley,

<u>Research Protocol MREC/99/0/78: Third International Stroke Trial - Thrombolysis for acute ischaemic stroke.</u>

Thank you for submitting the amendments previously requested by the Multi Centre Research Ethics Committee for Scotland.

The Chairman of the Multi-Centre Research Ethics Committee for Scotland, acting on delegated authority, has agreed to confirm the ethical approval granted by the Committee on 14 October 1999 on the understanding that you will follow the protocol as agreed and subject to the attached conditions of approval.

The following items were reviewed and the versions shown below were approved:

Protocol [IST-3 dated September 1999]	[✓]
Protocol amendments [reference number/version/date]	[ ]
Data sheet	[✓]
Subject information sheet [version 1.1 October 1999]	[✓]
Information sheet for Relatives and Carers [version 1.1 October 1999]	[✓]
Subject consent form [IST3]	[✓]
Witnessed consent form [IST3]	[✓]
Waiver of consent form	[✓]
GP letter 1, IST-3 information	[✓]
GP letter 2, Pre-follow-up	[✓]
GP letter 2, proforma	[✓]
Draft follow-up questionnaire [15/9/99]	[✓]
Draft follow-up questionnaire – patient in hospital [15/9/99]	[✓]
Methods of initial recruitment to study	[✓]
Compensation arrangements for subjects	[✓]
Payments to researchers	[✓]
Provision of expenses for subjects	[✓]

The following members were present at the meeting on 14 October 1999:

Professor C Gillis (Chairman)(Consultant in Public Health Medicine)
Dr N Anderson (Statistician)
Professor T MacDonald (Clinical Pharmacologist)
Dr G Masterton (Consultant Psychiatrist)
Mrs J Munro (Professions Allied to Medicine)
Professor P Peattie (Nurse)
Mr P Rogers (Consultant Surgeon)
Mrs M Shotter (Statistician)
Rev. T Tait (Lay)
Dr R Taylor (General Practitioner)

The Multi-Centre Research Ethics Committee for Scotland raised the following issues which have now been satisfactorily resolved:

- concern that it may be difficult to ensure that all centres would have consistently high standard of ethical consent
- the impact on private medical insurance of recruiting patients unable to give informed consent
- clarify how local/community councils would be contacted for consent for patients with no known relatives
- the patient information sheet should:
  - have a simple study title
  - have a version number and date
  - indicate the number of patients participating in the study
  - mention that placebo fluid would also be given by IV
  - mention who was sponsoring or funding the study
  - emphasise more clearly the reason for 6 hour time limit to decide
  - reassure patients that the researchers would not be paid
  - under "How is treatment given" refer to the position of the controls
  - mention the follow-up period
  - include under "Uncertainty principle (absence of proof)" the information provided for Q15
  - include a reference to compensation along the following lines:
    - "If you are harmed by taking part in this research project, there are no special compensation arrangements. If you are harmed due to someone's negligence, then you may have grounds for a legal action but you may have to pay for it. Regardless of this, if you wish to complain about any aspect of the way you have been approached or treated during the course of this study, the normal National Health complaints mechanisms may be available to you."
- provide an information sheet for relatives
- the consent form should ask if the GP could be contacted.

You should enclose a copy of this letter along with Annexe D of the Application Form and a copy of the Protocol (incorporating the amendments) together with any other relevant documentation to any local researcher who is going to participate in the project. It remains your responsibility to ensure that the Local Researchers obtain appropriate Local Research Ethics Committee approval and management approval.

Yours sincerely,

Walter Hunter

WALTER HUNTER
Administrator
Multi-Centre Research Ethics Committee for Scotland
cc: Mrs Tricia Cracknell, Stroke Association
