# Supplementary material for: The third international stroke trial (IST-3) of thrombolysis for acute ischaemic stroke
Source: Trials. 2008 Jun 17;9:37. doi: 10.1186/1745-6215-9-37 (PMC2442584; doi:10.1186/1745-6215-9-37)
Supplement: Additional file 3 — Multicentre Research Ethics approval. Multicentre Research Ethics approval. [file 1745-6215-9-37-S3.pdf]

Multi-Centre Research Ethics Committee for Scotland

Secretariat
Deaconess House
148 Pleasance
Edinburgh
EH8 9RS
Telephone 0131 536 9067
Fax 0131 536 9346
www.corec.org.uk

NHS SCOTLAND

Dr Richard Lindley
Dept of Clinical Neurosciences
Bramwell Dott Building
Western General Hospital
Edinburgh EH4 2XU

Date: 4th November 2003
Your Ref.:
Our Ref.: MREC/99/0/78

Enquiries to: Dorothy Garrow
Direct Line: 0131 536 9067
Email: dorothy.garrow@lhb.scot.nhs.uk

Dear Dr Lindley

MREC/99/0/78: Third International Stroke Trial: Thrombolysis for acute ischaemic stroke.

Thank you for your recent letter enclosing an amendment to the above study. The Amendments Sub-Committee of the Multi-Centre Research Ethics Committee for Scotland has reviewed the documents outlined below:

Final Approved Papers:
Covering letter dated 27th October 2003
Amendment Approval Request form
Protocol Version 1.9 dated 27th October
Report of the Date Monitoring Committee.
Randomisation Notepad
Patient treatment and Monitoring Record
6 month follow up Questionnaire
18th month follow up Questionnaire

The Sub-Committee has agreed the amendment.

This letter, together with details of the amendment submitted to the Amendments Sub-Committee, should be sent to the LRECs involved in the review of the study for information only.

Chairman Professor Kennedy Lees
Vice-Chairman Dr Geroge Masterton

Yours sincerely

Dorothy Garrow

DOROTHY GARROW
MREC Administrative Officer

cc: Mrs Tricia Cracknell
Stroke Association
Whitecross Street
London
EC1Y 8JJ

Cc: Professor Peter Sandercock
Principal Investigator
Division of Clinical Neurosciences
University of Edinburgh
Bramwell Dott Building
Western General Hospital
Crewe Road
Edinburgh
