# Supplementary material for: The third international stroke trial (IST-3) of thrombolysis for acute ischaemic stroke
Source: Trials. 2008 Jun 17;9:37. doi: 10.1186/1745-6215-9-37 (PMC2442584; doi:10.1186/1745-6215-9-37)
Supplement: Additional file 4 — Multicentre Research Ethics approval of amendment. Multicentre Research Ethics approval. [file 1745-6215-9-37-S4.pdf]

Multi Centre Research Ethics
Committee for Scotland

Secretariat

Deaconess House
148 Pleasance
Edinburgh
EH8 9RS
Tel: 0131 536 9067
Fax: 0131 536 9346
e-mail: Dorothy.Garrow@lhb.scot.nhs.uk

NHS
SCOTLAND

28th November 2006

29 NOV 2006

Professor P.A.G Sandercock
Department of Clinical Neurosciences
Bramwell Dott Building
Western General Hospital
Crewe Road
Edinburgh
EH4 2XU

Dear Professor Sandercock

Study title: Third International Stroke Trial: Thrombolysis for acute ischaemic stroke.

REC reference: MREC/99/0/78
Protocol number: IST1399/1.92
EudraCT number: 2004-000238-36

The above amendment was reviewed at the meeting of the Scottish MREC Amendments Sub- Committee held on 28th November 2006

The members of the Committee present gave a favourable ethical opinion of the amendment on the basis described in the notice of amendment form and supporting documentation. However the committee would prefer that the following wording is changed from:

1st Paragraph, 4th line down starting with "If not we will arrange for a second scan as part of the study which will expose you to a small amount of extra X Rays".

To Read:
"If not we will arrange for a second scan as part of the study which will be equivalent to **X** months background radiation".

The documents reviewed and approved at the meeting were:

Substantial Amendment Form
Summary of proposed amendment
Patient Information Leaflet – Version 1.5 dated 9/11/2006
Legal Representative Information leaflet – Version 1.5 dated 9/11/2006

Chairman Professor Kennedy Lees
Vice-Chairman Dr George Masterton

The members of the Ethics Committee who were present at the meeting are listed on the attached sheet.

Research governance approval

All investigators and research collaborators in the NHS should notify the R&D Department for the relevant NHS care organisation of this amendment and check whether it affects research governance approval of the research.

Statement of compliance

[CTIMPs only]

This Committee is recognised by the United Kingdom Ethics Committee Authority under the Medicines for Human Use (Clinical Trials) Regulations 2004, and is authorised to carry out the ethical review of clinical trials of investigational medicinal products.

The Committee is fully compliant with the Regulations as they relate to ethics committees and the conditions and principles of good clinical practice.

[All studies]

The Committee is constituted in accordance with the Governance Arrangements for Research Ethics Committees (July 2001) and complies fully with the Standard Operating Procedures for Research Ethics Committees in the UK.

REC reference number: MREC99/0/78 Please quote this number on all correspondence

Yours sincerely

Dorothy Garrow

Dorothy Garrow
Committee Co-ordinator

Chairman Professor Kennedy Lees
Vice-Chairman Dr George Masterton

Scottish MREC Sub-Committee

Meeting Held on 28th November 2006
Members Present:
Chairman

Dr George Masterton

Dr Alan Munro
